# Immune profiling in multiple sclerosis: a single-center study of 65 cytokines, chemokines, and related molecules in cerebrospinal fluid and serum

**DOI:** 10.3389/fimmu.2023.1200146

**Published:** 2023-06-13

**Authors:** Klaus Berek, Angelika Bauer, Dagmar Rudzki, Michael Auer, Robert Barket, Anne Zinganell, Magdalena Lerch, Livia Hofer, Astrid Grams, Paulina Poskaite, Sebastian Wurth, Thomas Berger, Franziska Di Pauli, Florian Deisenhammer, Harald Hegen, Markus Reindl

**Affiliations:** ^1^ Department of Neurology, Medical University of Innsbruck, Innsbruck, Austria; ^2^ VASCage Research Centre on Vascular Ageing and Stroke, Innsbruck, Austria; ^3^ Department of Neuroradiology, Medical University of Innsbruck, Innsbruck, Austria; ^4^ Department of Neurology, Medical University of Graz, Graz, Austria; ^5^ Department of Neurology, Medical University of Vienna, Vienna, Austria; ^6^ Comprehensive Center for Clinical Neurosciences and Mental Health, Medical University of Vienna, Vienna, Austria

**Keywords:** cytokine, chemokine, multiple sclerosis, prognosis, biomarker

## Abstract

**Introduction:**

The understanding of the pathophysiology of multiple sclerosis (MS) has evolved alongside the characterization of cytokines and chemokines in cerebrospinal fluid (CSF) and serum. However, the complex interplay of pro- and anti-inflammatory cytokines and chemokines in different body fluids in people with MS (pwMS) and their association with disease progression is still not well understood and needs further investigation. Therefore, the aim of this study was to profile a total of 65 cytokines, chemokines, and related molecules in paired serum and CSF samples of pwMS at disease onset.

**Methods:**

Multiplex bead-based assays were performed and baseline routine laboratory diagnostics, magnetic resonance imaging (MRI), and clinical characteristics were assessed. Of 44 participants included, 40 had a relapsing–remitting disease course and four a primary progressive MS.

**Results:**

There were 29 cytokines and chemokines that were significantly higher in CSF and 15 in serum. Statistically significant associations with moderate effect sizes were found for 34 of 65 analytes with sex, age, CSF, and MRI parameters and disease progression.

**Discussion:**

In conclusion, this study provides data on the distribution of 65 different cytokines, chemokines, and related molecules in CSF and serum in newly diagnosed pwMS.

## 1 Introduction

Measuring the inflammatory activity in people with MS (pwMS) can be done through contrast-enhancing lesions (CELs) on brain magnetic resonance imaging (MRI) scans (1, 2). However, CELs mainly reflect the blood–brain barrier breakdown-associated invasion of immune cells and are less common in progressive MS. Despite a decline in T and B lymphocytes and plasma cells in progressive MS lesions (3–5) compared to early MS lesions (3, 6), there is no difference in levels of B-cell (sCD21) and T-cell (sCD27) biomarkers in the CSF of people with a relapsing–remitting, primary progressive or secondary progressive MS (7). These findings support the role of inflammatory processes in progressive disease that are not captured by CELs (3, 7, 8) and further highlight the need for more effective and prognostic biomarkers for this complex, polygenic, and chronic disease, especially as current immunomodulatory treatments become less effective as MS progresses (9–12).

Studies have shown that cytokines play a significant role in the autoimmune nature of MS, with both Th1 (e.g., IFN-γ, IL-2, IL-12) and Th17 (e.g., IL-17, IL-21) cell-driven inflammation being linked to the disease (13–16). In recent years, also many other inflammatory cytokines were described to be elevated in the blood or CSF of pwMS compared to controls. Due to the close proximity to inflammatory lesions in MS, CSF biomarkers were believed to be the most promising ones and intensively focused on in research. In a large recently performed meta-analysis of 226 studies on 13,526 pwMS and 8,428 controls including 26 CSF-based and 37 blood-based cytokines, especially the B-lymphocyte chemoattractant (BLC or CXCL13) was found the most promising in CSF to distinguish between pwMS and various controls. Levels decreased during therapy, reflecting its potential role in disease pathophysiology and thus highlighting the importance to perform cytokine analysis in untreated pwMS (16).

As peripheral inflammation is also believed to play a crucial part in MS pathogenesis (17), as blood is less invasive to collect than lumbar puncture and as the sensitivity of various analytical methods improved over time, blood-based biomarkers in MS have various advantages. One of the most promising serum biomarkers are neurofilament light chain (sNfL) levels, as abundant evidence showed their potential to reflect ongoing inflammation-driven neuroaxonal damage (18). Furthermore, also some cytokines in serum showed promising results to distinguish pwMS from controls, for instance IL-23, IL-2, or IL-17. However, the relationship between these cytokines and disease progression or relapse activity remains unclear as results from different studies have been inconsistent (16). Despite the lack of a single biomarker that accurately predicts disease course in all pwMS, a combination of laboratory, imaging, and clinical parameters is currently used in clinical practice to guide treatment decisions. There is an urgent need for dynamic biomarkers that can provide more detailed information about intrathecal inflammation and specific cell processes.

The main aim of our study was to quantify concentrations of 65 cytokines, chemokines, and related molecules in paired serum and CSF samples of newly diagnosed and untreated pwMS. Our secondary study aims subsumed identifying possible links between these concentrations and clinical or MRI progression and their correlation with routine laboratory parameters.

## 2 Materials and methods

### 2.1 Study design

In a prospective research project with a nationwide scope in Austria, people diagnosed with multiple sclerosis (pwMS) were recruited at the Clinical Department of Neurology, Medical University of Innsbruck, with the aim of identifying biomarkers that are associated with disease progression, with a specific emphasis on progression into primary or secondary progressive disease course. People newly diagnosed with MS according to the 2017 revised McDonald Criteria (19), aged 18–65, with diagnostic routine lumbar puncture performed at the Clinical Department of Neurology, Medical University of Innsbruck, were eligible for inclusion to the study. Exclusion criteria were disease-modifying treatment at lumbar puncture, corticosteroid treatment within the last 6 months prior to lumbar puncture, and a cerebrospinal fluid (CSF) red blood cell (RBC) count >500/μl (20).

The study consisted of a single simultaneous collection of CSF and serum samples and regular clinical and radiological investigations. The first was performed during baseline visit for routine diagnostic purpose in people with suspected MS. Furthermore, at baseline, clinical, demographic, and radiological data were assessed. CSF as well as serum samples were stored immediately at -80°C until further use. Study participants underwent regular clinical examinations at least every 6 months.

The herein collected parameters included the disability status as indicated by the Expanded Disability Status Scale (EDSS), disease history, including date and type of first symptom, relapse history, and treatment history. Additionally, data of sex, age, and ethnicity of the person were collected at baseline. Relapses were defined as subacute neurological deteriorations typical for demyelinating events occurring in the absence of infections or fever and with a time gap of at least 30 days to the last relapse (21). We defined EDSS progression as a worsening of EDSS scores of ≥1.5 points in patients with an EDSS score at baseline of 0, of ≥1.0 point in those with EDSS baseline scores between 1.0 and 5.5 and of ≥0.5 points, if patients had baseline scores above 5.5. All EDSS values had to be confirmed after 6 months.

Radiological data subsumed number of cerebral and spinal lesions showing hyperintensity in T2-weighted images and number of cerebral and spinal lesions showing contrast enhancement (CE) in T1-weighted images. We defined radiological progression as an increase in T2-hyperintense or in CE lesions in the follow-up MRI scan compared to the baseline MRI scan.

### 2.2 Primary and secondary study aims

The primary aim of our study was to quantify CSF and serum concentrations of a broad panel of cytokines, chemokines, and related molecules in the abovementioned population of pwMS. Secondary study aims were to reveal possible associations of these concentrations and the occurrence of relapses, EDSS progression, or MRI progression as defined above. Furthermore, we aimed to investigate correlations between these concentrations and routine laboratory parameters.

### 2.3 Magnetic resonance imaging

Magnetic resonance imaging was performed centrally at the Department of Neuroradiology, Medical University of Innsbruck, Austria, at a field strength of 1.5 or 3 T. Scans with axial slices with a maximum thickness of 5 mm were obtained. The assessment of the above mentioned radiographic characteristics (T2 hyperintense lesions, CE lesions) was performed by raters (AG and PP) experienced in MRI diagnostics and blinded for any further information (clinical, demographic, and laboratory data).

### 2.4 Routine laboratory diagnostics

Routine laboratory data were assessed at the Neuroimmunology Laboratory, Department of Neurology, Medical University of Innsbruck, Austria, and performed for routine diagnostic purpose. It comprises CSF counts of white blood cells (WBC) and RBC, CSF, and serum albumin, immunoglobulin (Ig) G, A and M in CSF and serum, and oligoclonal IgG bands (OCB), which were analyzed as previously published (22–26). By dividing CSF albumin/serum albumin, the albumin quotient (Q-Alb) was calculated. To define elevated Q-Alb values, a cutoff was calculated by an age-dependent formula, as reported previously (27). The WBC was defined as elevated with values ≥5/µl, and an elevation of RBC was defined with a count above 500/µl. The IgG index was calculated according to published formulars, and a cutoff of >0.7 was used in this study (28, 29).

### 2.5 Measurements of serum and CSF cytokines, chemokines, and related molecules

Levels of 65 cytokines, chemokines, and related molecules were measured using commercially available immunoassays (Thermo Fisher Scientific, Waltham, MA, USA; Immune Monitoring 65-Plex Human ProcartaPlex panel, cat. #EPX650-10065-901) according to the manufacturer’s instructions. Therefore, samples stored at -80°C were thawed on ice and then used immediately. Magnetic beads were washed with 25 µl universal assay buffer, and 25 µl of standards, 25 µl of undiluted serum, or undiluted CSF samples were added and incubated for 120 min at room temperature (RT) on a shaker at 500 rounds per minute (rpm). After washing, 25 µl of a detection antibody mixture was added and incubated for 30 min at RT on a shaker. After washing twice, 50 µl streptavidin-phycoerythrin solution was incubated for 30 min on a shaker at RT and washed afterwards. Then, 120 µl reading buffer was added into each well, and after a 5-min incubation period while shaking at RT fluorescence, intensity was measured on a Luminex MAGPIX instrument (Software: xPONENT 4.2 and ProcartaPlex Analyst 1.0). In samples where analytes were not detectable or their concentrations exceeded the highest standard, the values of the lowest or highest standards were used for statistical analyses. A concise list of all measured 65 molecules was published by Bauer et al. (30) and is included in all figures.

The ratios of CSF to serum concentrations for all evaluated cytokines, chemokines, and related molecules were determined by dividing the respective CSF concentrations by their corresponding serum concentrations.

### 2.6 Ethics declaration

This study was approved by the ethical committee of the Medical University of Innsbruck (ethical approval number: AN3041 257/4.8 393/5.4 (4319a)), and all participants gave written informed consent prior to inclusion.

### 2.7 Statistics

For statistical analyses, the software packages GraphPad Prism (GraphPad Software, La Jolla, California, USA; version 9) and IBM SPSS (IBM SPSS Statistics, Armonk, New York, USA; version 28) were used. Within-group comparisons between CSF and serum samples were calculated using the Wilcoxon signed-rank test for paired samples. P-values were corrected for multiple comparisons using a false discovery rate significance criterion of 1% based on the Benjamini, Krieger, and Yekutieli correction.

The PANTHER (Protein ANalysis THrough Evolutionary Relationships) classification system with the PANTHER overrepresentation test (released 20221013) and the GO gene ontology database (31) were used for annotation of analytes according to their association with PANTHER GO SLIM biological processes (32, 33).

Correlations were evaluated by Spearman’s rank correlation tests and Spearman’s rho correlation coefficients were classified according to Cohen (34) as weak (<-0.2 to -0.5 or >0.2 to 0.5) or moderate (<-0.5 to -0.8 or >0.5 to 0.8) (34).

The association of CSF:serum ratios of cytokines/chemokines with sex and clinical or MRI progression was analyzed using the Mann–Whitney test. Effect sizes were calculated according to Cohen (1988) (34) and classified as weak or moderate as described above:


$$R=\;\frac{z}{\sqrt{n}}$$

The statistical power of correlation coefficients was calculated using G*Power 3.1 software for 44 participants and α = 0.05 (35). Only correlation coefficients of<-0.3 or >0.3 (statistical power >0.52) were considered as relevant.

## 3 Results

Overall, a total of 44 predominantly female (64%) pwMS with a median age of 31 years were included in the study. At baseline, 40 showed a relapsing–remitting (RR) and four a primary progressive (PP) disease course. The median disease duration, defined as time between first symptom and baseline visit, was 41 days. The vast majority of pwMS (77%) showed a monofocal disease onset. According to the inclusion criteria, no study participant received DMT. Patients showed a “minimal disability” as indicated by a median EDSS score of 1.0. At baseline, in all pwMS, cerebral MRI was performed with a median of 10 hyperintense lesions in T2-weighted images, while in 37 people with a spinal MRI, a median of one T2 hyperintense lesion was revealed. In total, the criteria of “Dissemination in space” (DIS) was fulfilled in 43 people. A total of 26 people showed at least one CE lesion whether in cerebral or in spinal MRI and therefore fulfilled the criteria of “Dissemination in time” (DIT) at baseline.

CSF analysis was available in all 44 pwMS with a median WBC count of 7.5/µl, resulting in an elevated WBC in 30 (68.2%) pwMS. The median RBC count was 0, with no CSF sample exceeding an RBC count of 500/µl. The median QAlb was 4.3, and blood–brain-barrier dysfunction as indicated by elevated QAlb was detected in only two (4.6%) pwMS. The median IgG index was 0.84, in 36 people (81.8%) the IgG index was above 0.7, and all participants showed positive OCB.

At the median follow-up visit of 25 months after baseline, one individual with a previously diagnosed RR form of MS progressed into a secondary progressive (SP) MS. Eight pwMS experienced a progression in their EDSS score, and 14 experienced at least one relapse event. As a result, 11 pwMS required initiation of a moderately effective DMT and 15 required a highly effective DMT by the last follow-up. **Tables 1**, **2** provide an overview of clinical, demographic, radiographic, and routine laboratory data.

**Table 1:** Demographic and cerebrospinal fluid characteristics of study participants at baseline and follow-up.

	Baseline	Last follow-up
Number of patients	44	44
Age (years)^1^	31 (19-58)	32 (21-60)
Sex (females)^2^	28 (63.6)	28 (63.6)
Disease course		
Relapsing remitting^2^	40 (90.9)	39 (88.6)
Primary progressive^2^	4 (9.1)	4 (9.1)
Secondary progressive^2^	0 (0.0)	1 (2.3)
Disease duration (months)^1,a^	1 (0-340)	28 (10-370)
Follow-up duration (months)^1,b^	n.a.	25 (9-39)
First symptom		
Monofocal^2^	34 (77.3)	n.a.
Optic neuritis^2^	11 (25.0)	n.a.
Brainstem or cerebellum^2^	12 (27.3)	n.a.
Myelitis^2^	14 (31.8)	n.a.
Other^2^	15 (34.1)	n.a.
EDSS^1^	1.0 (0.0-4.0)	1.0 (0.0-7.0)
EDSS progression until last FU^1^	n.a.	8 (18.2)
Relapse activity until last FU^2^	n.a.	14 (31.8)
DMT total^2^		
None^2^	44 (100)	18 (40.9)
Moderately effective^2,c^	0 (0)	11 (25.0)
Highly effective^2,d^	0 (0)	15 (34.1)
White blood cells (/µL)^1^	7.5 (0-45)	n.a.
Red blood cells (/µL)^1^	0 (0-362.5)	n.a.
CSF total protein (mg/dL)^1^	39.5 (21.0-106.0)	n.a.
Q-Alb^1^	4.3 (2.0-16.2)	n.a.
IgG-index^1^	0.84 (0.47-2.62)	n.a.
CSF IgG OCB positive^2^	44 (100)	n.a.

Data are shown as ^1^median (range), ^2^number (percentage). ^a^Time between first symptom and respective study visit (baseline, last follow-up). ^b^Time between baseline and last clinical follow-up. ^c^Subsumes dimethyl fumarate (n = 10) and teriflunomide (n = 1). ^d^Subsumes natalizumab (n = 4), ocrelizumab (n = 8), ofatumumab (n = 1), and fingolimod (n = 2).

CSF, cerebrospinal fluid; DMT, disease-modifying treatment; EDSS, Expanded Disability Status Scale; FU, follow-up; Ig, immunoglobulin; OCB, oligoclonal bands; Q-Alb, albumin quotient; RBC, red blood cell; TP, total protein; WBC, white blood cell. n.a. = not applicable.

**Table 2:** Radiological characteristics of study participants at baseline and last radiological follow-up.

Radiological characteristics	Baseline	Last radiological follow-up
Total number of patients	44	33 (75.0)
Disease duration until MRI (months)^1,a^	1 (0-340)	17 (6-112)
MRI follow-up duration (months)^1,b^	n.a.	14.0 (6-49)
Field strength 3 T^1^	10 (21.3)	34 (72.3)
Cerebral MRI performed^1^	44 (100)	33 (100)
Cerebral T2 lesions^2^	10 (2-60)	12 (3-50)
Cerebral CE lesions^2^	0 (0-8)	0 (0-2)
Spinal MRI performed^1^	40 (85.1)	0 (0)
Spinal T2 lesions^2^	1 (0-7)	n.a.
Spinal CE lesions^2^	0 (0-3)	n.a.
Total T2 lesions^2^	12 (3-61)	n.a.
Total CE lesions^2^	1 (0-8)	n.a.
MRI progression	n.a.	18 (54.6)

Data are shown as ^1^number (percentage), ^2^median (range). ^a^Time between first symptom and respective study visit (baseline, last follow-up). ^b^Time between baseline and last MRI follow-up.

CE, contrast-enhancing; MRI, magnetic resonance imaging; T2 lesion, hyperintense lesion in T2-weighted MRI images. n.a. = not applicable.

### 3.1 Cytokine, chemokine, and related biomarker concentrations in CSF and serum

**Figure 1:**
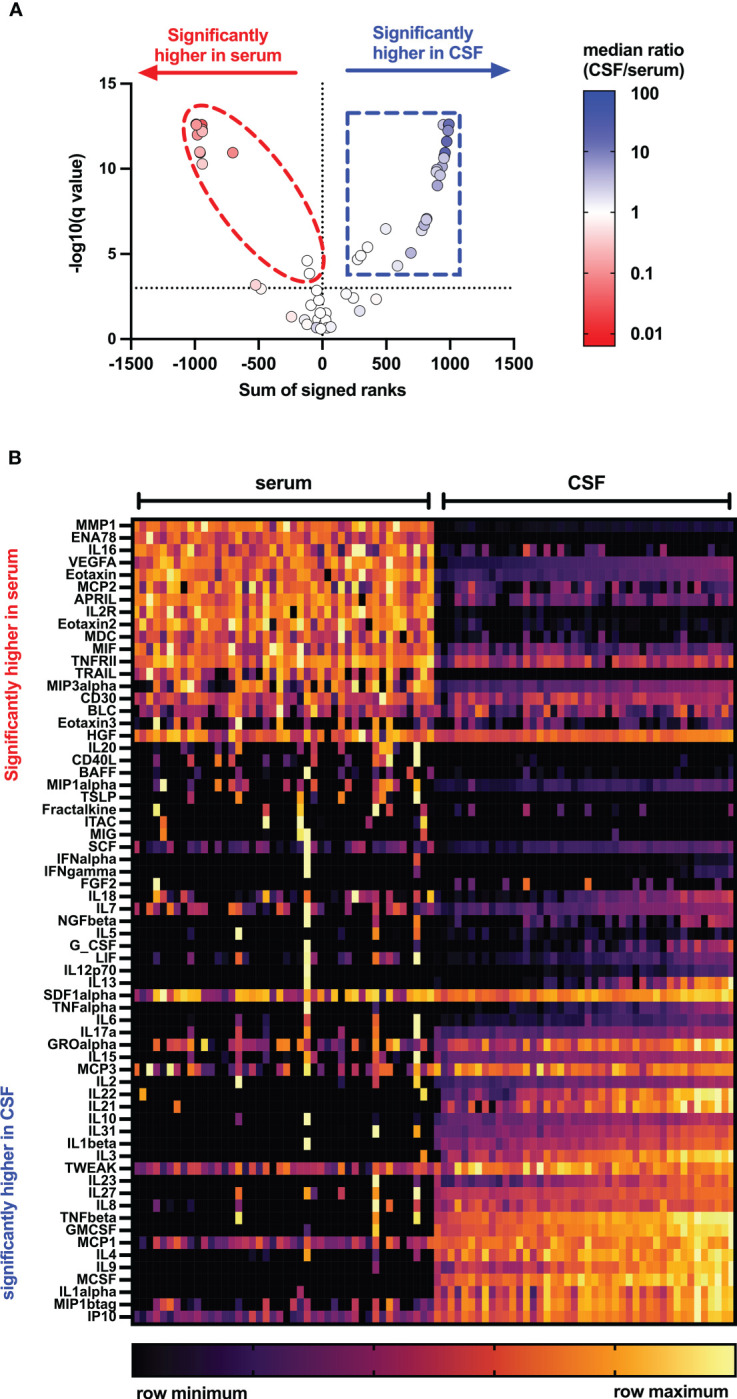
Distribution of cytokines, chemokines, and related molecules in serum and CSF of people with MS. Volcano plots **(A)** and heat map **(B)** showing levels of 65 cytokines, chemokines, and related molecules in serum (left) and cerebrospinal fluid (right) of pwMS using Mann–Whitney tests after two-stage step-up (Benjamini, Krieger, and Yekutieli) and FDR of 1% correction for multiple comparisons. APRIL, a proliferation-inducing ligand; BAFF, B-cell activation factor; BLC, B-lymphocyte chemoattractant; CD, cluster of differentiation; CSF, cerebrospinal fluid; ENA, epithelial neutrophil-activating peptide; FGF, fibroblast growth factor; GCSF, granulocyte colony-stimulating factor; GMCSF, granulocyte-macrophage colony-stimulating factor; GRO, growth-regulated oncogene; HGF, hepatocyte growth factor; IFN, interferon; IL, interleukin; IP, interferon-γ-induced protein; ITAC, interferon-inducible T-cell α-chemoattractant; LIF, leukemia inhibitory factor; MCP, monocyte chemoattractant protein; MCSF, macrophage colony-stimulating factor; MDC, macrophage-derived chemokine; MIF, macrophage migration inhibitory factor; MIG, IFN-gamma-induced monokine; MIP, macrophage inflammatory protein; MMP, matrix metalloproteinase; NGF, nerve growth factor; SCF, stem cell factor; SDF, stromal cell-derived factor; TNF, tumor necrosis factor; TRAIL, TNF-related apoptosis-inducing ligand; TSLP, thymic stromal lymphopoietin; TWEAK, tumor necrosis factor-like weak inducer of apoptosis; VEGF, vascular endothelial growth factor.

**Figure 2:**
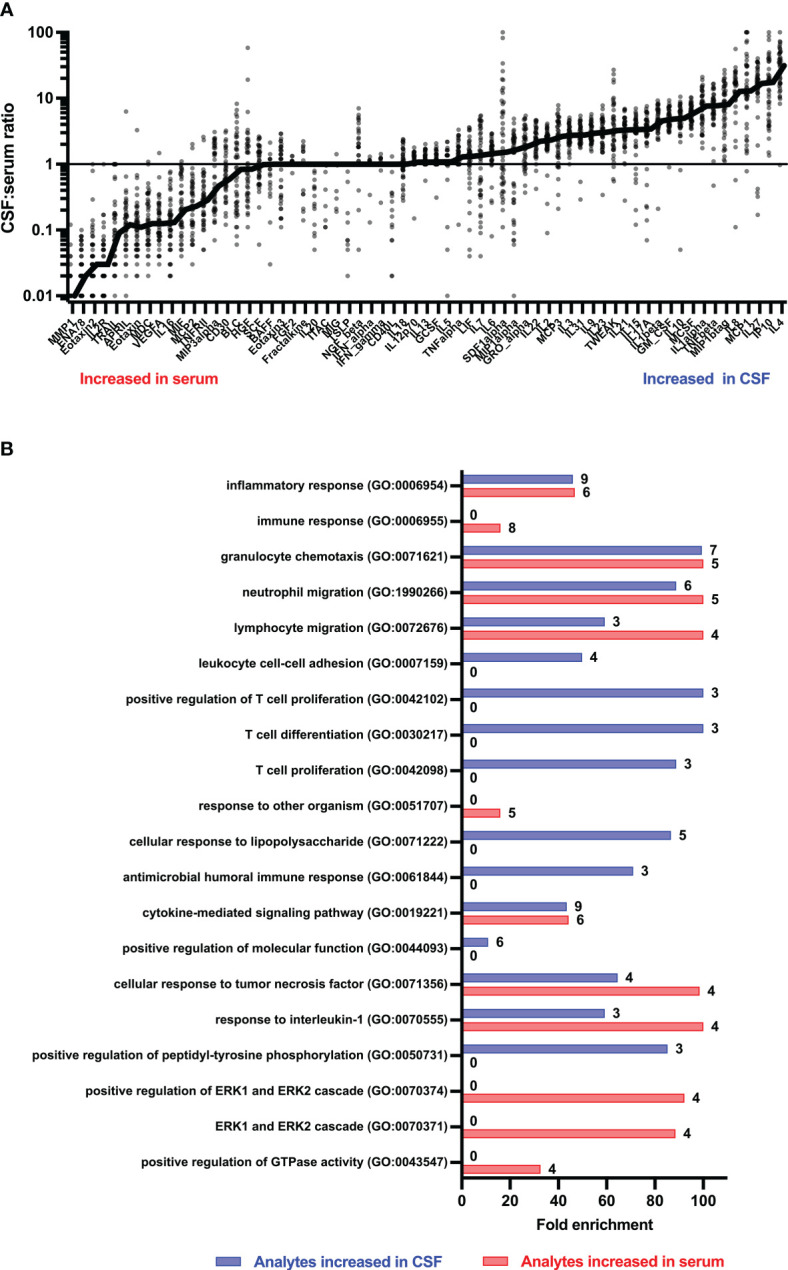
CSF/serum ratio for 65 cytokines, chemokines, and related molecules in people with MS. **(A)** Heatmap showing individual study participants’ CSF/serum ratio for all measured cytokines, chemokines, and related molecules. p-Values were calculated using Wilcoxon matched-pair signed-rank test with two-stage step-up (Benjamini, Krieger, and Yekutieli) correction comparing cytokine levels of serum with CSF using a false discovery rate of 1%. Only those cytokines, chemokines, and related molecules with a significant p-value<0.05 were highlighted. **(B)** Significantly deregulated analytes were annotated to biological functions using the PANTHER classification system with the PANTHER overrepresentation test (released 20221013) and the GO gene ontology database (DOI: 10.5281/zenodo.6799722 Released 2022-07-01). APRIL, a proliferation-inducing ligand; BAFF, B-cell activation factor; BLC, B-lymphocyte chemoattractant; CD, cluster of differentiation; CSF, cerebrospinal fluid; ENA, epithelial neutrophil-activating peptide; ERK, extracellular signal-regulated kinase; FGF, fibroblast growth factor; GCSF, granulocyte colony-stimulating factor; GMCSF, granulocyte-macrophage colony-stimulating factor; GRO, growth-regulated oncogene; HGF, hepatocyte growth factor; IFN, interferon; IL, interleukin; IP, interferon-γ-induced protein; ITAC, interferon-inducible T-cell α-chemoattractant; LIF, leukemia inhibitory factor; MCP, monocyte chemoattractant protein; MCSF, macrophage colony-stimulating factor; MDC, macrophage-derived chemokine; MIF, macrophage migration inhibitory factor; MIG, IFN-gamma-induced monokine; MIP, macrophage inflammatory protein; MMP, matrix metalloproteinase; NGF, nerve growth factor; SCF, stem cell factor; SDF, stromal cell-derived factor; TNF, tumor necrosis factor; TRAIL, TNF-related apoptosis-inducing ligand; TSLP, thymic stromal lymphopoietin; TWEAK, tumor necrosis factor-like weak inducer of apoptosis; VEGF, vascular endothelial growth factor.

**Figures 1**, **2** display CSF/serum ratios of all analytes. A significant difference between CSF and serum concentrations was observed for 51 cytokines, chemokines, and related molecules. In 33 of these analytes, the absolute concentration was higher in CSF, while in 19 it was higher in serum. No significant differences were found in the concentrations of 14 analytes in serum compared to CSF. Median concentrations and CSF:serum correlations of all cytokines, chemokines, and related molecules are shown in **Supplementary Tables 1, 2**. A subset of cytokines, chemokines, and related molecules that were deregulated in both serum and CSF were biologically linked to functions related to the inflammatory response, granulocyte chemotaxis, and neutrophil and lymphocyte migration. In contrast, some cytokines or chemokines that showed significant increases in CSF were associated with positive regulation during T-cell proliferation, differentiation, cellular response to lipopolysaccharides, and humoral immune response against microbes.

### 3.2 Association of peripheral and CSF inflammation with routine laboratory characteristics

**Figure 3:**
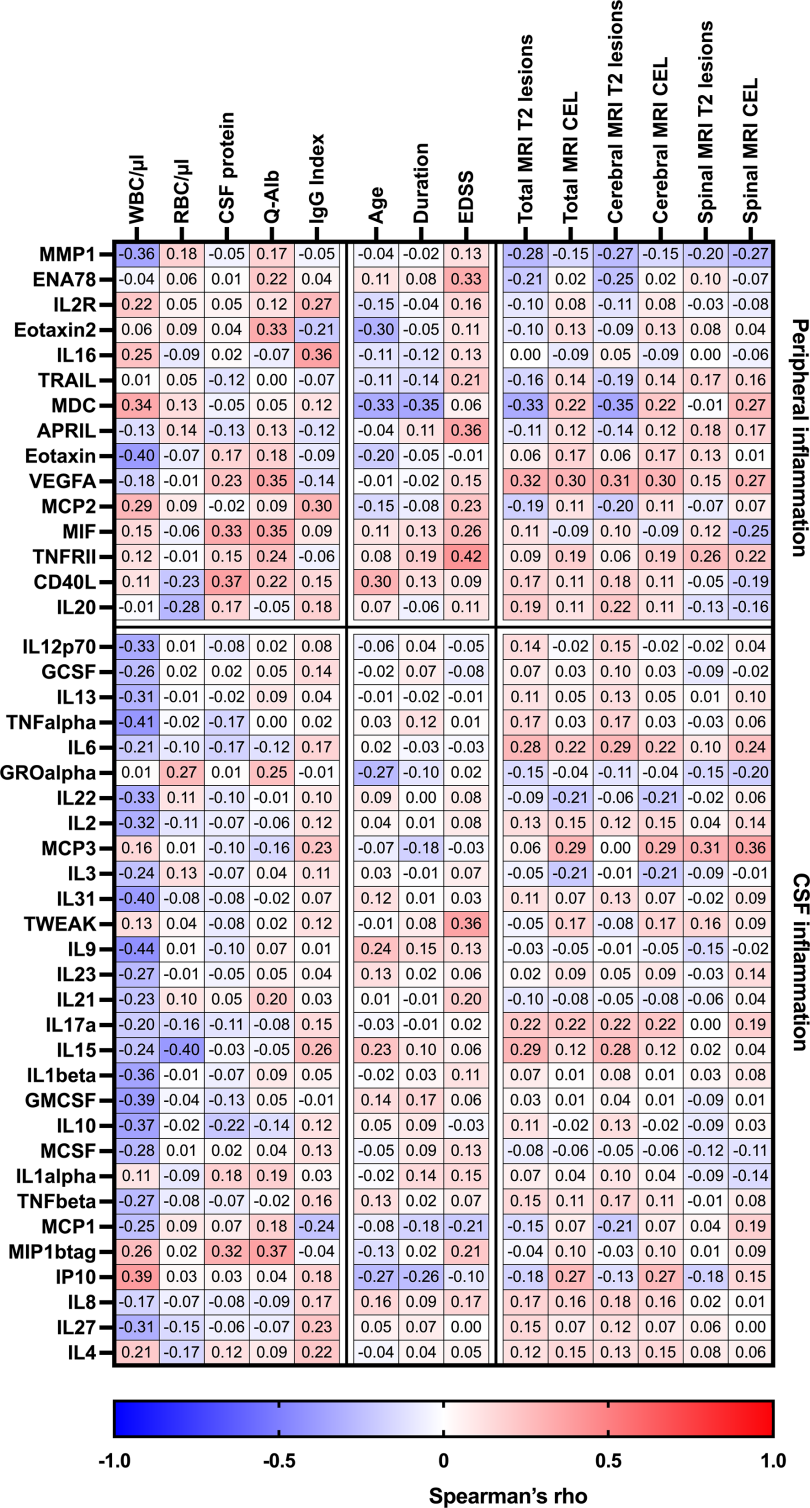
Heatmap of Spearman’s correlation coefficients between CSF/serum ratios of analytes increased in serum (peripheral inflammation) or CSF (CSF inflammation) and routine CSF laboratory characteristics in pwMS. Spearman correlation coefficients are depicted as colors ranging from blue to red, presenting low up to high correlation between serum cytokine or chemokine concentrations and baseline routine CSF laboratory characteristics. APRIL, a proliferation-inducing ligand; CD, cluster of differentiation; CEL, contrast-enhancing lesions, CSF, cerebrospinal fluid; EDSS, Expanded Disability Status Scale; ENA78, epithelial neutrophil-activating peptide-78; GCSF, granulocyte colony-stimulating factor; GMCSF, granulocyte-macrophage colony-stimulating factor; GRO, growth-regulated oncogene; IL, interleukin; Ig, immunoglobulin; IP, interferon-gamma-induced protein; MCP, monocyte chemoattractant protein; MCSF, macrophage colony-stimulating factor; MDC, macrophage-derived chemokine; MIF, macrophage migration inhibitory factor; MIP, macrophage inflammatory protein; MMP, matrix metalloproteinase; MRI, magnetic resonance imaging; Q-Alb, CSF albumin/serum albumin; RBC, red blood cell count; TNF, tumor necrosis factor; TRAIL, TNF-related apoptosis-inducing ligand; TWEAK, tumor necrosis factor-like weak inducer of apoptosis; VEGF, vascular endothelial growth factor; WBC, white blood cell count; T2 lesion, hyperintense lesion in T2-weighted MRI images.

Cytokines or chemokines classified as related with peripheral inflammation (increased serum concentrations compared to CSF) had an overrepresentation of chemokines. They were found to at least moderately correlate with many routine laboratory characteristics and clinical parameters as well as with some MRI baseline findings, as shown in **Table 3**; **Figure 3**. For instance, this study could find a positive correlation between MIF (R = 0.33) and CD40L (R = 0.37) with CSF protein count. Additionally, MIF showed a positive correlation with Q-Alb. High levels of IL-16 were associated with a higher IgG index. A negative correlation was observed between age and levels of CD40L, eotaxin-2, and MDC. Furthermore, MDC negatively correlated with the total number of MRI cerebral lesions and disease duration.

**Table 3:** Spearman correlations of analytes associated with peripheral and/or CSF inflammation.

	Peripheral inflammation	CSF inflammation
WBC/µl	Eotaxin (ρ=-0.40↓), MDC (ρ=-0.34↓), MMP1 (ρ=-0.36↓)	GM-CSF (ρ=-0.39↓), IL1ß (ρ=-0.36↓), IL2 (ρ=-0.32↓), IL9 (ρ=-0.44↓), IL10 (ρ=-0.37↓), IL12p70 (ρ=-0.33↓), IL13 (ρ=-0.30↓), IL22 (ρ=-0.33↓), IL27 (ρ=-0.31↓), IL31 (ρ=-0.40↓), IP10 (ρ=0.39↑), TNFα (ρ=-0.41↓)
RBC/µL		IL15 (ρ=-0.40↓)
CSF protein	CD40L (ρ=0.37↑), MIF (ρ=0.33↑)	MIP1btag (ρ=0.32↑)
Q-Alb	Eotaxin-2 (ρ=0.33↑), MIF (ρ=0.35↑), VEGF-A (ρ=0.35↑)	MIP1btag (ρ=0.37↑)
IgG index	IL16 (ρ=0.36↑)	
Age	CD40L (ρ=-0.30↓), eotaxin-2 (ρ=-0.30↓), MDC (ρ=-0.33↓)	
Duration	MDC (ρ=-0.35↓)	
EDSS	APRIL (ρ=0.36↑), ENA78 (ρ=0.33↑), TNFRII (ρ=0.42↑)	TWEAK (ρ=0.36↑)
Total MRI T2 lesions	MDC (ρ=-0.33↓), VEGF-A (ρ=0.32↑)	
Cerebral MRI T2 lesions	MDC (ρ=-0.35↓), VEGF-A (ρ=0.31↑)	
Spinal MRI T2 lesions		MCP3 (ρ=0.31↑)
Spinal MRI CEL		MCP3 (ρ=0.36↑)

APRIL, a proliferation-inducing ligand; CD40L, cluster of differentiation 40 ligand; CEL, contrast-enhancing lesions; CSF, cerebrospinal fluid; EDSS, Expanded Disability Status Scale; ENA78, epithelial-derived neutrophil-activating protein 78; GM-CSF, granulocyte macrophage colony stimulating factor; Ig, immunoglobulin; IL, interleukin; MCP, monocyte chemoattractant protein; MDC, macrophage-derived chemokine; MIF, macrophage migration inhibitory factor; MIP, macrophage inflammatory protein; MMP, matrix metalloproteinase; MRI, magnetic resonance imaging; Q-Alb, albumin quotient; RBC, red blood cell; T2 lesion, hyperintense lesion in T2-weighted MRI images; TNF, tumor necrosis factor; TWEAK, tumor necrosis factor-like weak inducer of apoptosis; VEGF-A, vascular endothelial growth factor A; WBC, white blood cell.

Only statistically significant (p< 0.05) Spearman correlation coefficients ρ > 0.3 (moderate-strong effect sizes, statistical power >50%) are shown.↑ Indicate positive, ↓ indicate negative correlations.

When analyzing CSF inflammation (defined as CSF:serum ratio >1), various cytokines and chemokines were associated with routine laboratory characteristics. Notably, negative correlations were found between CSF white blood cell count and several CSF cytokines (GM-CSF, IL1β, IL2 IL9, IL10, IL12p70, IL13, IL22, IL27, IL31, IP10, TNFα). Additionally, TWEAK was the only analyte that showed at least moderate associated with an increase in EDSS, while MCP3 was correlated with the occurrence of spinal MRI lesions.

### 3.3 Effect of peripheral or CSF inflammation on clinical and MRI disease progression

With regard to peripheral inflammation, CD40L (R = 0.31↑) was the only protein that showed a positive association with a progressive disease course at follow-up and MDC (R = 0.30↑) was the only molecule associated with an EDSS progression at the last follow-up. Furthermore, higher baseline serum levels of APRIL were predictive of a MRI progression after 1 year (R = 0.40↑).

In the CSF, high baseline levels of GROα (R = 0.30↑) were linked with higher relapse activity at the last follow-up. Also, two CSF cytokines, IL-2 and IL8, had a strong impact on MRI progression after 1 year. At baseline, a dissemination in time on MRI was linked to higher CSF levels of IL22 (R = 0.30↑).

A higher EDSS score at baseline and a longer disease duration were both predictors for EDSS progression after 1 year, and a higher age at baseline was an additional predictor for a progressive MS disease course. Older age at baseline and more spinal T2 lesions, especially when these are contrast-enhancing lesions, were predictors of MRI progression after 1 year.

**Table 4**; **Figure 4** provide more detailed information on the effect sizes (R) of the CSF:serum ratios of all cytokines, chemokines, and related molecules on clinical and MRI data at onset and last follow-up.

**Table 4:** Association of analytes with peripheral and/or CSF inflammation, and clinical/CSF/MRI parameters with sex, clinical presentation at onset, and clinical and MRI progression at last follow-up or 1-year follow-up.

	Peripheral inflammation	CSF inflammation	Clinical/CSF/MRI parameters
Females	ENA78 (R=-0.47↓), Eotaxin2 (R=-0.42↓), TRAIL (-0.38↓), APRIL (R=-0.52↓), IL20 (R=-0.32↓)	TWEAK (R=-0.38↓)	
Multifocal onset		IL1α (R=0.39↑), IP10 (R=0.35↑), IL4 (R=0.47↑)	
Relapse activity last FU		GROα (R=0.30↑)	Age BL (R=0.45↑), FU time (R=0.40↑)
Progressive MS last FU	CD40L (R=0.31↑)		Age BL (R=0.43↑), duration BL (R=0.49↑), EDSS BL (R=0.56↑)
EDSS progression Y1			Duration BL (R=0.46↑), EDSS BL (R=0.38↑)
EDSS progression last FU	MDC (R=0.30↑)		Duration BL (R=0.44↑)
MRI DIS BL	CD40L (R=0.30↑), IL20 (R=0.30↑)		
MRI DIT BL		IL22 (R=0.30↑)	WBC/µl (R=0.31↑), total MRI T2 lesions (R=0.31↑), total MRI CEL (R=0.84↑), cerebral MRI CEL (R=0.65↑), spinal MRI CEL (R=0.53↑)
MRI DIS and DIT BL			Total MRI T2 lesions (R=0.33↑), total MRI CEL (R=0.84↑), cerebral MRI CEL (R=0.65↑), spinal MRI CEL (R=0.53↑)
MRI progression Y1	APRIL (R=0.40↑)	IL2 (R=0.31↑), IL8 (R=0.41↑)	Age BL (R=0.35↑), spinal MRI T2 lesions (R=0.30↑), spinal MRI CEL (R=0.32↑)

APRIL, a proliferation-inducing ligand; BL, baseline; CEL, contrast-enhancing lesions; CSF, cerebrospinal fluid; DIS, dissemination in space; DIT, dissemination in time; EDSS, Expanded Disability Status Scale; ENA78, epithelial-derived neutrophil-activating protein 78; FU, follow-up; GRO, melanoma growth stimulating activity; IL, interleukin; IP10, interferon-gamma-induced protein 10; MDC, macrophage-derived chemokine; MRI, magnetic resonance imaging; T2 lesion, hyperintense lesion in T2-weighted MRI images; TRAIL, tumor necrosis factor-related apoptosis inducing ligand; TWEAK, tumor necrosis factor-like weak inducer of apoptosis; WBC, white blood cell; Y1: 1-year follow-up.

Groups were analyzed using Mann–Whitney U test, and only statistically significant (p< 0.05) differences with effect sizes R > 0.3 (moderate-strong effect sizes, statistical power >50%) are shown.↑ Indicate positive, ↓ indicate negative correlations.

**Figure 4:**
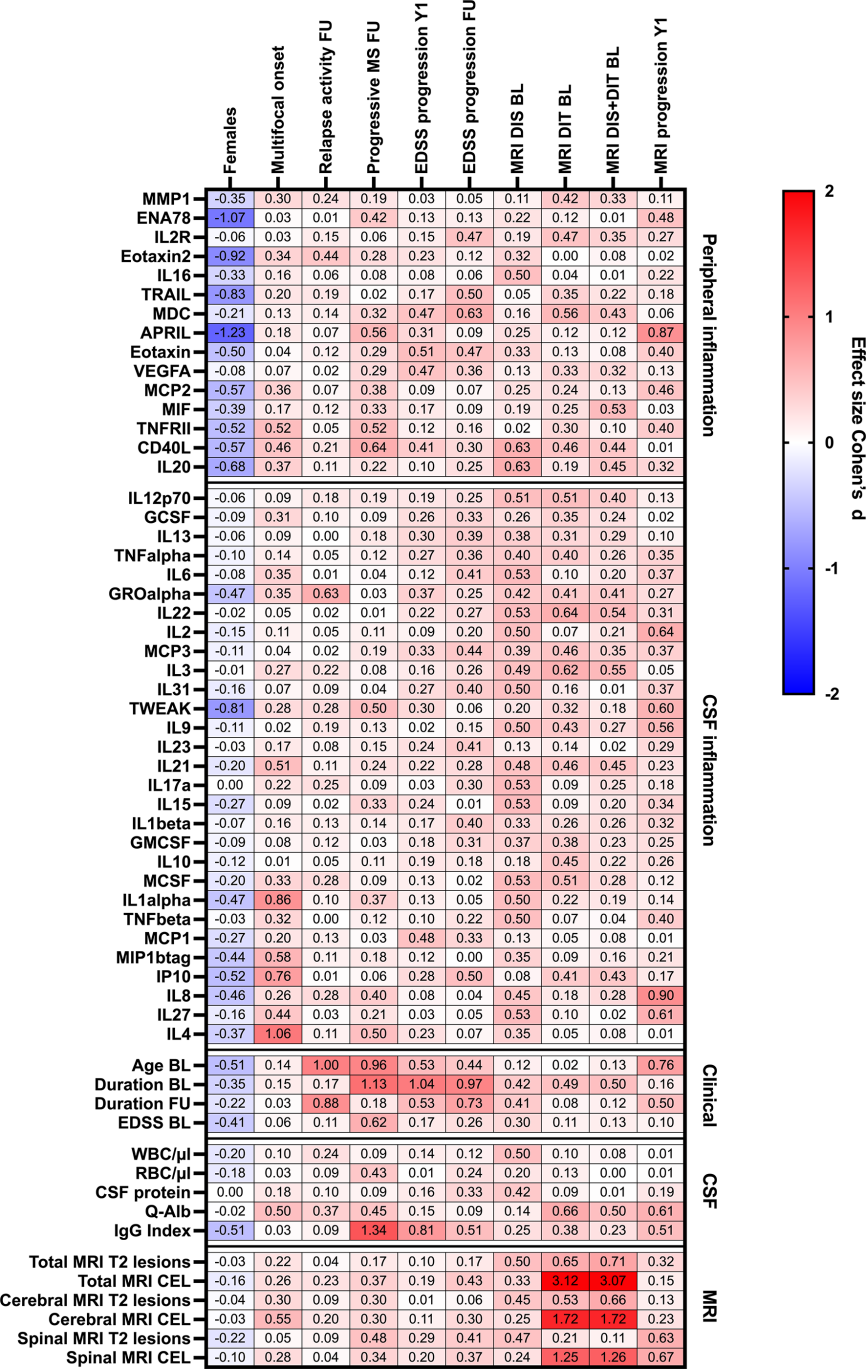
Heatmap of effects sizes R of CSF/serum ratios of analytes increased in serum (peripheral inflammation) or CSF (CSF inflammation) and sex, clinical and MRI presentation at onset, and clinical and MRI progression. Spearman correlation coefficients are depicted as colors ranging from blue to red, presenting low up to high correlation between serum cytokine or chemokine concentrations and baseline routine CSF laboratory characteristics. APRIL, a proliferation-inducing ligand; BL, baseline; CD, cluster of differentiation; CEL, contrast-enhancing lesions; CSF, cerebrospinal fluid; DIS, dissemination in space; DIT, dissemination in time; EDSS, Expanded Disability Status Scale; ENA78, epithelial neutrophil-activating peptide-78; FU, follow-up; GCSF, granulocyte colony-stimulating factor; GMCSF, granulocyte-macrophage colony-stimulating factor; GRO, growth-regulated oncogene; Ig, immunoglobulin; IL, interleukin; IP, interferon-gamma induced protein; MCP, monocyte chemoattractant protein; MCSF, macrophage colony-stimulating factor; MDC, macrophage-derived chemokine; MIF, macrophage migration inhibitory factor; MIP, macrophage inflammatory protein; MMP, matrix metalloproteinase; MRI, magnetic resonance imaging; Q-Alb: CSF albumin/serum albumin; RBC, red blood cell count; T2 lesion, hyperintense lesion in T2-weighted MRI images; TNF, tumor necrosis factor; TRAIL, TNF-related apoptosis-inducing ligand; TWEAK, tumor necrosis factor-like weak inducer of apoptosis; VEGF, vascular endothelial growth factor; WBC, white-blood cell count; Y1, 1-year follow-up.

## 4 Discussion

In the present study, we characterized the immune profile of pwMS by measuring a broad panel of 65 cytokines, chemokines, and related molecules in CSF and serum. Here, we could demonstrate significantly higher levels of 15 cytokines, chemokines, and related molecules in serum compared to CSF and higher levels of 29 of these proteins vice versa.

The pathophysiology of MS is a complex interplay of mechanisms leading to demyelination as well as axonal and astrocytic damage (36, 37). A key player in this interplay is inflammation, driven by autoreactive B and T cells directed against CNS patterns. Once activated, they can infiltrate the CNS leading to inflammation and tissue damage (37). Altered cytokine and chemokine metabolism paves the way for priming, proliferation, and activation of immune cells as well as for their invasion to the CNS (38, 39). In context of many contradictory results on cytokine analysis in pwMS, influenced by different control groups, different analytical methods, or the use of different statistical analysis, we provide further detailed information on concentrations of a broad spectrum of these analytes and their distribution in the body fluids serum and CSF.

Especially Th1 cytokines (IL-1β, IL-2, IL-12, TNFα, IFNγ) have been described as important drivers for inflammation in pwMS (14, 40–42). Our findings of significantly increased levels of IL-1β, IL-2, and TNFα in CSF compared to serum support this role of Th1-driven inflammation in the pathogenesis of MS. Among the CD4+ T-cells, also Th17 cells were found to be crucial in disease pathophysiology (43), secreting IL-17A, IL-21, and IL-22, all three significantly increased in CSF in our cohort. Findings of higher cytokine concentrations in CSF are in line with prior studies revealing intrathecal inflammation as the main driver of disease progression and disability in MS (8).

However, also chemokines trigger inflammation, by supporting lymphocyte, macrophage, and monocyte infiltration in lesions and plaques (44, 45). They are produced and secreted locally in tissues, induce and activate leukocyte adhesion molecules, establish chemotactic concentration gradients and thus, by inducing proteolytic enzymes, support an opening of the BBB, and finally mediate leukocyte trafficking into the CNS (46–48). These functions are also reflected by our gene ontology and PANTHER classification system analysis. In our study, especially in the CSF, increased cytokines and chemokines were found to have a role in T-cell differentiation, leukocyte cell–cell adhesion, neutrophil migration, and cytokine-mediated immune response and are in general involved in inflammatory response and granulocyte chemotaxis. For example, was MCP-1 one of the most concentrated chemokines in CSF compared to serum in pwMS in our cohort, which is in line with others (49). This chemokine takes a potent role in the recruitment of macrophages, monocytes, activated T cells, and natural killer cells to the CNS and site of inflammation. Producing cells are mainly endothelial cells, astrocytes, macrophages, and microglia (50). MCP-1 has been shown to be crucial in the induction of acute experimental autoimmune encephalomyelitis, an animal model of MS (51), and CSF levels are described to be dependent on disease activity, with lower CSF levels in active MS than in stable MS (49, 52), although this was not seen in our cohort.

On the other hand, also shown by gene ontology and PANTHER classification system analysis, many in serum increased cytokines and chemokines have a role in inflammatory response, immune response, migration of neutrophils, lymphocytes, and granulocytes and interestingly in the ERK1 and ERK2 cascade. ERK1/2 are the final components of the mitogen-activated protein kinase (MAPK) signaling cascade, involved in different cellular functions such as cell proliferation, differentiation, adhesion, migration, and survival (53). In microglia, researchers could show an overactivation of MAPK, causing demyelination of oligodendrocytes, which is a known hallmark of MS (54).

A further interesting observation are significantly higher levels of macrophage and granulocyte colony stimulating factors (M-CSF, G-CSF, and GM-CSF) in CSF than in serum. Recently, a lively research interest concerning the role of the cytokine GM-CSF has evolved (55). For instance, a pathologically expanded T-cell population has been defined, which was characterized by production of specific cytokines including GM-CSF, IL-2, and TNFα (56). Furthermore, it has been demonstrated that GM-CSF plays a key role for the CNS invasion of phagocytes and consequently for neuroinflammation and that the GM-CSF-producing T-cell subset was regulated by IL-23 and IL-1β (57). Finally, lymphocytes in general and the pathologically expanded MS-specific T-cell population in particular have demonstrated its potential to migrate effectively against a SDF1α gradient (56). Deduced from our data, such a gradient may be hypothesized between CSF and serum, displayed by a slightly higher concentration of SDF1α in CSF than in serum.

Among others, APRIL and BAFF are known to be involved in B-cell survival and proliferation. The importance of B-cell activity in the pathogenesis of MS is emphasized by the success of B-cell depleting therapies (11, 58). As expected, this typically in plasma samples detected protein APRIL revealed significantly lower CSF than serum levels in this study. This is consistent with a study on 30 treatment-naïve pwMS measuring lower levels of APRIL and BAFF in CSF compared to serum. Nevertheless, it was demonstrated by comparing pwMS to healthy controls that for both cytokines, CSF levels were elevated in pwMS but not serum levels (59). On the contrary, another study showed conflicting results with higher BAFF levels in serum and plasma of pwMS compared to healthy controls (60, 61). Reasons for different cytokine levels between studies may be explained by the influence of disease-specific treatment (62, 63) or short-term methylprednisolone treatment (60, 61), thus underlining the importance of studies on treatment-naïve pwMS.

Taking into account our correlation analyses between serum and CSF cytokine and chemokine levels, the positive correlation of, e.g., CD40L and IL-23, has to be pointed out. This may be of special interest, as blood samples are less invasive and easier to collect and therefore could potentially be taken serially. Nevertheless, it has to be pointed out that these correlations are only weak and only for CD40L and IL-23 were they found to be statistically significant. This is also in line with a recent study investigating CSF and serum levels of 36 cytokines and chemokines in MS patients, revealing a significant correlation of CSF and serum levels in only three cases (namely, CCL23, also known as MIP-3; CCL27 and IL-6) (64).

In our study, disability worsening determined by EDSS worsening correlated with serum MDC levels, while inflammatory activity detected by relapse activity did so with CSF GROα levels. The macrophage-derived chemokine MDC is a chemokine produced by monocyte-derived dendritic cells and macrophages and is upregulated by the Th2-related cytokine IL-4 or IL-5 (65). This chemokine acts as a chemoattractant for the migration of CC chemokine receptor 4 (CCR4)-expressing cells, which include Th2/Treg cells, to sites of inflammation (43). In pwMS, decreased serum levels of MDC have been described compared to healthy individuals (66) and especially female pwMS were reported to have lower serum levels (66). In our cohort, we could also see differences between females and males, as especially female sex was associated with lower levels of some cytokines in serum (ENA-78, Eotaxin-2, TRAIL, APRIL, IL-20) or CSF (TWEAK), although this might be influenced by our relatively low sample size in general. GROα, or also known as CXCL1, is a chemokine crucial in inflammatory processes, and specifically in models for MS, it was shown to have both neuroprotective but also neurotoxic properties as it contributes to neurodegeneration (67, 68). For pwMS, it was described that harmful properties of GROα may dominate, as one study found a positive correlation of plasma levels with progressing EDSS, thus reflecting clinical disability (69). On the contrary, increased levels of GROα were also described in the CSF of pwMS compared to controls, however, irrespectively of the presence of gadolinium-enhancing lesions (70).

In our study, a progressive course of MS was found to be associated with higher levels of serum CD40L but not with cytokine markers of CSF inflammation. Although in our study only very few participants had a progressive course of MS, our finding is consistent with those of another study which observed higher serum soluble CD40L levels in people with active MS compared to those with an inactive disease (71). CD40L is the typical ligand for CD40, also known as CD40–CD40L dyad. This dyad functions as an immune checkpoint regulator and promotes both humoral and cellular immune responses, leading to an upregulation of cytokine receptors and other inflammatory or costimulatory molecules (72, 73). Since CD40L is expressed not only on T cells and platelets, but during inflammation also on B cells, dendritic cells, macrophages, and others (74), research has been conducted to investigate the use of an anti-CD40L monoclonal antibody as a potential new therapeutic target. A phase I clinical trial was already conducted, showing no serious adverse events over an 18-week study period (72, 75). Unfortunately, in a clinical trial of Crohn’s disease patients, a severe case of thromboembolism occurred using an anti-CD40L monoclonal antibody (76). However, not only was CD40L found to be associated with a higher probability of a progressive disease course at follow-up but also higher age at baseline and a higher EDSS level at baseline. This is in line with others, describing preexisting disability and older age as risk factors for disability accumulation and progressive disease in MS (77).

MRI data provided in our cohort has to be assessed with caution, as exclusively patients at first diagnosis have been included who usually presented due to clinical episodes of high disease activity. Probably, this may have also influenced MRI scans, especially in terms of presence and number of CEL. For instance, in our cohort only MDC and VEGFA correlated with cerebral T2 lesions while MCP3 did so in terms of spinal T2 and CE lesions, while IL-22 correlated with fulfilling the radiological criteria of dissemination in time at baseline. Prima vista, this seems to contradict recent findings of higher levels of IL-12p40, TNFα, TNFβ, and IL-10 in patients with lesional inflammatory activity defined as presence of CEL or clinical relapses (78). Nevertheless, this may be explained by the abovementioned possible recruiting bias in our cohort.

There are some limitations of this study. For the broad panel of 65 measured chemokines and cytokines, our study had probably a too low number of participants and was therefore underpowered. Consequently, results that were not significant in statistical tests cannot be interpreted in contrast to those that show significant differences between groups. Furthermore, to avoid random findings due to the measurement of such a high number of proteins, we corrected for multiple testing. Moreover, our radiological follow-up period was not as long as the clinical follow-up duration and both were relatively short. A further limitation of our study may be seen in the small number of people with a primary progressive disease course at the last follow-up. Indeed, it has been demonstrated by others that higher inflammatory cytokine profiles were described in people with RRMS compared to those with a progressive disease course, indicating a higher level of inflammatory activity (79, 80). Therefore, further investigations with higher sample sizes and longer follow-up durations are needed, in order to be able to stratify according to MS disease courses.

## 5 Conclusion

In this study, we provide data of concentrations and distribution of 65 cytokines, chemokines, and related molecules in CSF and serum of people suffering from MS. These findings may also provide an important basis for the selection of cytokines or chemokines for further studies.

## Data availability statement

The original contributions presented in the study are included in the article/**Supplementary Material**. Further inquiries can be directed to the corresponding author.

## Ethics statement

The studies involving human participants were reviewed and approved by Ethical committee of the Medical University of Innsbruck (Innsbruck, Austria); ethical approval number: AN3041 257/4.8 393/5.4 (4319a. The patients/participants provided their written informed consent to participate in this study.

## Author contributions

KB: investigation, data curation, formal analysis, writing—original draft preparation; AB: investigation, data curation, formal analysis, writing—original draft preparation; DR: investigation, data curation, writing—reviewing and editing; MA: writing—reviewing and editing; RB: writing—reviewing and editing; AZ: writing—reviewing and editing; ML: writing—reviewing and editing; LH: writing—reviewing and editing; AG: investigation, writing—reviewing and editing; PP: investigation, writing—reviewing and editing; SW: writing—reviewing and editing; TB: writing—reviewing and editing; FP: writing—reviewing and editing; FD: writing—reviewing and editing; HH: writing—reviewing and editing; MR: conceptualization, supervision, visualization, data curation, formal analysis, writing—original draft preparation. All authors contributed to the article and approved the submitted version.
