# Supplementary material for: Immune profiling in multiple sclerosis: a single-center study of 65 cytokines, chemokines, and related molecules in cerebrospinal fluid and serum
Source: Front Immunol. 2023 Jun 13;14:1200146. doi: 10.3389/fimmu.2023.1200146 (PMC10294231; doi:10.3389/fimmu.2023.1200146)
Supplement: Supplementary file 1 [file Table_1.pdf]

Immune profiling in Multiple Sclerosis: A single center study of 65 cytokines, chemokines and related molecules in cerebrospinal fluid and serum

Klaus Berek[1]†, Angelika Bauer[1,2]†, Dagmar Rudzki[1,2], Michael Auer[1], Robert Barket[1], Anne Zinganell[1], Magdalena Lerch[1], Livia Hofer[1], Astrid Grams[3], Paulina Poskaite[3], Sebastian Wurth[4], Thomas Berger[5,6], Franziska Di Pauli[1], Florian Deisenhammer[1], Harald Hegen[1], Markus Reindl[1*]

Supplementary table 1. Median concentrations of 65 cytokines, chemokines and related molecules in serum and CSF of people with multiple sclerosis at baseline.

Molecule	median (range) serum concentration [pg/ml]	median (range) CSF concentration [pg/ml]	Molecule	median (range) serum concentration [pg/ml]	median (range) CSF concentration [pg/ml]
APRIL	1586.4 (44.1-6720.9)	164.4 (44.1-416.4)	IL-12p70	7.6 (7.6-53.8)	8.2 (7.6-13.7)
BAFF	5.7 (5.7-217.0)	5.7 (5.7-12.0)	IL-13	3.5 (3.5-6.9)	3.8 (3.5-7.1)
BLC	74.9 (11.0-1116.9)	45.3 (14.5-282.0)	IL-15	3.9 (3.9-172.0)	15.1 (7.7-27.0)
CD30	268.8 (58.4-1547.6)	200.1 (43.1-392.2)	IL-17A	23.3 (23.3-1930.2)	89.6 (48.8-176.2)
ENA-78	165.3 (37.1-2240.5)	3.7 (3.7-3.8)	IL-18	7.6 (7.0-39.3)	10.0 (7.0-18.8)
Eotaxin-2	143.1 (4.1-764.3)	4.7 (4.1-12.0)	IL-2	19.3 (19.3-382.8)	47.6 (30.2-79.4)
Eotaxin-3	1.5 (1.5-23.5)	2.1 (1.5-4.9)	IL-21	6.8 (6.8-24.9)	22.7 (6.8-47.1)
FGF-2	4.5 (4.5-7.0)	4.5 (4.5-6.0)	IL-22	19.9 (19.9-81.3)	44.8 (19.9-92.8)
Fractalkine	2.6 (2.6-17.9)	2.6 (2.6-5.8)	IL-23	16.2 (16.2-231.4)	50.8 (24.6-125.5)
IL-16	171.1 (24.5-785.4)	13.5 (13.5-146.1)	IL-27	14.9 (14.9-3175.2)	289.4 (135.2-601.3)
IL-2R	2529.9 (81.4-11373.4)	81.4 (81.4-134.9)	IL-3	23.1 (23.1-77.0)	64.7 (23.1-117.2)
IL-20	7.1 (7.1-139.3)	7.1 (7.1-7.1)	IL-31	12.3 (12.3-156.0)	35.3 (21.7-66.9)
I-TAC	9.7 (9.7-89.9)	9.7 (9.7-9.7)	IL-4	19.4 (19.4-750.5)	679.3 (159.4-2085.7)
MCP-2	7.2 (0.8-24.1)	1.5 (0.8-5.4)	IL-5	6.8 (6.8-535.5)	7.4 (6.8-14.7)
MCP-3	9.2 (4.6-49.7)	23.4 (7.7-41.6)	IL-6	5.2 (5.2-134.6)	8.5 (5.2-15.2)
MDC	178.9 (18.4-1023.6)	18.4 (18.4-65.9)	IL-7	1.4 (0.5-45.0)	1.8 (0.8-3.4)
MIF	125.0 (30.9-649.5)	29.4 (10.2-56.1)	IL-8	2.0 (2.0-339.6)	33.3 (8.4-111.7)
MIG	8.6 (8.6-46.0)	8.6 (8.6-12.2)	IL-9	10.7 (10.7-20.3)	31.8 (16.8-61.1)
TNFR2	87.2 (18.1-350.6)	30.7 (3.6-84.1)	IP-10	19.3 (2.5-141.3)	263.9 (46.9-2491.0)
TRAIL	127.1 (11.4-1278.2)	11.4 (11.4-11.4)	LIF	4.5 (4.5-250.1)	7.7 (4.5-17.5)
TSLP	6.7 (6.7-286.2)	6.7 (6.7-6.7)	MCP-1	65.6 (3.7-447.0)	544.9 (256.0-5678.2)
TWEAK	795.0 (126.6-2794.5)	2296.9 (544.2-7887.5)	M-CSF	63.4 (63.4-63.4)	386.2 (173.1-651.2)
NGFβ	4.0 (4.0-172.2)	4.5 (4.5-28.2)	MIP-1α	2.4 (2.4-301.9)	5.6 (3.7-11.9)
Eotaxin	42.6 (1.6-277.2)	4.5 (2.4-11.3)	MIP-1btag	3.1 (3.1-43.5)	41.4 (9.6-110.8)
G-CSF	9.9 (9.9-31.8)	10.7 (9.9-20.4)	MIP-3α	95.8 (16.5-398.2)	39.7 (24.8-73.5)
GM-CSF	13.1 (13.1-91.3)	62.8 (39.9-121.4)	MMP-1	1328.3 (64.2-19400.0)	8.4 (5.0-15.0)
GROα	4.9 (2.4-16.0)	8.8 (4.4-19.5)	CD40L	4.7 (4.7-261.7)	4.7 (4.7-7.6)
HGF	217.3 (4.9-1626.9)	175.6 (77.2-502.0)	SCF	4.3 (2.4-75.0)	4.5 (2.6-7.4)

IFNα	4.8 (4.8-37.1)	4.8 (4.8-6.3)	SDF-1α	5044.5 (38.8-21110.3)	4144.7 (1183.5-13504.2)
IFNγ	10.7 (10.7-40.4)	10.7 (10.7-15.3)	TNFα	4.9 (4.9-59.8)	6.8 (4.9-16.4)
IL-1α	1.6 (1.6-5.9)	12.6 (2.8-33.3)	TNFβ	6.1 (6.1-83.4)	47.7 (23.8-100.8)
IL-1β	3.4 (3.4-85.8)	15.9 (8.1-31.7)	VEGF-A	363.5 /62.9-1661.3)	42.8 (16.7-101.8)
IL-10	2.4 (2.4-232.6)	12.0 (5.2-25.3)			

Abbreviations:

APRIL: a proliferation-inducing ligand; BAFF: B cell activation factor; BLC: B lymphocyte chemoattractant; CD: cluster of differentiation; CSF: cerebrospinal fluid; ENA: epithelial neutrophil-activating peptide; FGF: fibroblast growth factor; GCSF: granulocyte colony-stimulating factor; GMCSF: granulocyte-macrophage colony-stimulating factor; GRO: growth-regulated oncogene; HGF: hepatocyte growth factor; IFN: interferon; IL: interleukin; IP: interferon-γ-induced protein; ITAC: interferon-inducible T cell α-chemoattractant; LIF: leukemia inhibitory factor; MCP: monocyte chemoattractant protein; MCSF: macrophage colony-stimulating factor; MDC: macrophage-derived chemokine; MIF: macrophage migration inhibitory factor; MIG: IFN-gamma-induced monokine; MIP: macrophage inflammatory protein; MMP: matrix metalloproteinase; NGF: nerve growth factor; SCF: stem cell factor; SDF: stromal cell-derived factor; TNF: tumor necrosis factor; TRAIL: TNF-related apoptosis-inducing ligand; TSLP: thymic stromal lymphopoietin; TWEAK: tumor necrosis factor-like weak inducer of apoptosis; VEGF: vascular endothelial growth factor

Supplementary table 2. Correlation of cytokines and chemokines between serum and CSF of people with multiple sclerosis at baseline.

Molecule	r_s	p-value	Molecule	r_s	p-value
APRIL	0.05	0.76	IL-12p70	-0.02	0.87
BAFF	0.21	0.18	IL-13	0.02	0.91
BLC	-0.08	0.58	IL-15	0.19	0.23
CD30	-0.10	0.53	IL-17A	0.06	0.70
ENA-78	-0.03	0.87	IL-18	0.13	0.41
Eotaxin-2	0.25	0.10	IL-2	0.11	0.48
Eotaxin-3	0.07	0.63	IL-21	-0.24	0.12
FGF-2	0.28	0.06	IL-22	-0.03	0.85
Fractalkine	0.10	0.50	**IL-23**	**0.30**	**0.04**
IL-16	0.19	0.22	IL-27	0.18	0.24
IL-2R	0.02	0.89	IL-3	0.20	0.20
IL-20	n.a.		IL-31	0.26	0.09
I-TAC	n.a.		IL-4	0.20	0.20
MCP-2	0.05	0.73	IL-5	0.09	0.56
MCP-3	0.19	0.21	IL-6	0.01	0.94
MDC	-0.04	0.82	IL-7	-0.21	0.17
MIF	-0.19	0.22	IL-8	0.21	0.16
MIG	-0.05	0.73	IL-9	0.18	0.24
TNFR2	0.06	0.70	IP-10	0.17	0.28
TRAIL	n.a.		LIF	0.28	0.07
TSLP	n.a.		MCP-1	0.18	0.25
TWEAK	0.16	0.31	M-CSF	n.a.	
NGFβ	0.15	0.34	MIP-1α	0.07	0.67
Eotaxin	0.09	0.57	MIP-1β	0.17	0.26
G-CSF	0.03	0.82	MIP-3α	0.15	0.32
GM-CSF	0.13	0.38	MMP-1	0.01	0.97
GROα	0.20	0.20	**CD40L**	**0.34**	**0.02**
HGF	0.01	0.99	SCF	-0.07	0.67
IFNα	0.09	0.56	SDF-1α	0.07	0.66

IFNγ	0.14	0.36	TNFα	0.10	0.54
IL-1α	0.10	0.51	TNFβ	0.17	0.26
IL-1β	0.08	0.59	VEGF-A	0.12	0.42
IL-10	0.04	0.80			

Legend:

Correlation coefficients (r_s) were analysed using Spearman's rho correlation test. Significant results with a two-sided p-value <0.05 were highlighted in bold.

Abbreviations:

APRIL: a proliferation-inducing ligand; BAFF: B cell activation factor; BLC: B lymphocyte chemoattractant; CD: cluster of differentiation; CSF: cerebrospinal fluid; ENA: epithelial neutrophil-activating peptide; FGF: fibroblast growth factor; GCSF: granulocyte colony-stimulating factor; GMCSF: granulocyte-macrophage colony-stimulating factor; GRO: growth-regulated oncogene; HGF: hepatocyte growth factor; IFN: interferon; IL: interleukin; IP: interferon-γ-induced protein; ITAC: interferon-inducible T cell α-chemoattractant; LIF: leukemia inhibitory factor; MCP: monocyte chemoattractant protein; MCSF: macrophage colony-stimulating factor; MDC: macrophage-derived chemokine; MIF: macrophage migration inhibitory factor; MIG: IFN-gamma-induced monokine; MIP: macrophage inflammatory protein; MMP: matrix metalloproteinase; NGF: nerve growth factor; SCF: stem cell factor; SDF: stromal cell-derived factor; TNF: tumor necrosis factor; TRAIL: TNF-related apoptosis-inducing ligand; TSLP: thymic stromal lymphopoietin; TWEAK: tumor necrosis factor-like weak inducer of apoptosis; VEGF: vascular endothelial growth factor
